# Environmental motivation or economic motivation? Explaining individuals’ intention to carry reusable bags for shopping in China

**DOI:** 10.3389/fpsyg.2022.972748

**Published:** 2022-11-03

**Authors:** Yong Li, Bairong Wang

**Affiliations:** ^1^School of Marxism, Shanghai Maritime University, Shanghai, China; ^2^School of Economics and Management, Shanghai Maritime University, Shanghai, China

**Keywords:** plastic ban awareness, social responsibility, environmental motivation, economic motivation, reusable bag using intention, China

## Abstract

To achieve satisfying effects of plastic ban policies, it is important to promote people’s intention to use green bags. Many studies have examined the antecedents of reducing plastic bag usage, but research regarding the influential factors of reusable bag usage is limited. Based on a survey of 532 respondents in China, a multiple linear regression model is constructed in this study to examine the determinants of individuals’ intention to carry reusable bags for shopping. Results show that plastic ban awareness, social responsibility, environmental motivation, and economic motivation significantly and positively affect consumers’ intention to use reusable bags for shopping. Of the two motivation factors, environmental motivation has a greater impact. More importantly, economic motivation positively moderates the relationship between environmental motivation and the intention to use reusable bags. This finding suggests a motivation “crowding-in” effect in predicting consumers’ intention to carry reusable bags for shopping. Results of this study also indicate that female, older, and richer people are more inclined to carry reusable bags for shopping. Implications for plastic crisis management are discussed.

## Introduction

Single-use plastics have become one of the most serious environmental problems around the world given the devastating damages they have caused in recent decades (Adam et al., 2020; Wang and Li, 2021). Consequently, how to reduce the negative impact of single-use plastics has attracted extensive global attention (Convery et al., 2007; He, 2012; Martinho et al., 2017; Li and Wang, 2021; Wang and Li, 2022). Many countries have launched plastic ban policies against the use of plastic bags and to promote the use of more environmentally friendly alternatives (Wang and Li, 2021), such as reusable bags (Madigele et al., 2017; Liu et al., 2021; Van et al., 2021). Carrying a reusable bag for shopping is identified as a pro-environmental behavior (Wang and Li, 2022). However, research regarding the determinants of reusable bag usage is quite limited compared with studies regarding other green behaviors (Convery et al., 2007; Afroz et al., 2017; Bharadwaj et al., 2020). To fill the potential research gap, this study is motivated to uncover the dynamics behind consumers’ intention to carry reusable bags for shopping.

With its huge population, China is one of the largest user of plastics in the world (Nyathi and Togo, 2020; Wang and Li, 2021). In 2020, the Chinese government introduced a plastic ban to mitigate the usage of plastics and promote the usage of green bags (Wang et al., 2021). Therefore, investigating the effectiveness of China’s plastic ban is crucial for addressing plastic pollution and providing plastic management implications worldwide. Except for plastic ban policies, social responsibility and different motivations are helpful in interpreting individuals’ green behavioral intention (Bénabou and Tirole, 2006; Gneezy et al., 2011; Bhattacharya, 2019). This study aims to investigate how plastic ban awareness, social responsibility, and motivations (environmental motivation and economic motivation) impact consumers’ intention to carry reusable bags for shopping in China. Specifically, this study also examines the interacting effect of environmental motivation and economic motivation on the intention to use reusable bags through the lens of motivation crowding theory (Ryan and Deci, 2000).

## Literature review and hypotheses

Typically, people’s pro-environmental behavior could either be intrinsically or extrinsically motivated. These two motivations could interact with each other when shaping people’s pro-environmental behavior (Xu et al., 2018; Authelet et al., 2021). For instance, the “crowding-out” and “crowding-in” theory explains the strengthening and weakening phenomenon of the two motivations, respectively (Deci et al., 1999; Bowles, 2008; Luck et al., 2012). Therefore, based on motivation crowding theory, we analyze the motivation dynamics behind consumers’ intention to carry reusable bags for shopping. Moreover, existing studies also show that contextual factors (e.g., policy) and people’s sense of social responsibility could exert significant influence on people’s pro-environmental behavior (Stern, 2000). Therefore, this study extends the theory structure by adding these two variables to increase the explaining power.

### The effects of plastic ban policies

An increasing number of studies show the positive effects of plastic ban policies in mitigating people’s plastic bag usage and in stimulating their reusable bag usage (Convery et al., 2007; He, 2012; Bharadwaj et al., 2021; Wang and Li, 2021). Convery et al. (2007) found that after the Irish government introduced a tax on single-use plastic bags in 2002, the consumption of plastic bags decreased by 94%. Likewise, Denmark applied a plastics tax on producers and retailers, producing a 66% drop in plastic bags (Dikgang et al., 2012). Similarly, in Portugal, the usage of plastic bags decreased by 74%, and the use of reusable bags increased by 61% after the plastic tax’s implementation (Martinho et al., 2017). The enforcement of China’s 2008 plastic ban policies generated a 49% reduction in the use of plastic bags (He, 2012). Moreover, it is reported that the awareness of plastic ban policies significantly promoted people’s reuse of old plastic bags for shopping in China (Li and Wang, 2021). Similar to recycling plastic bags, carrying reusable bags for shopping is also a positive response to plastic ban policies (Wang and Li, 2021). Thus, this study proposes the following hypothesis:

*Hypothesis 1 (H1)*: Plastic ban awareness has a positive impact on individuals’ intention to carry reusable bags for shopping.

### Social responsibility and environmentalism

Social responsibility means individuals are responsible for carrying out their civic duty, and an individual’s actions must benefit the society (Davila Gomez and Crowther, 2007). Typically, people with high social responsibility are altruistic and are usually concerned with the consequences of their actions and tend to plan for better future outcomes, including environmental outcomes (Borden and Francis, 1978; Hirsh, 2010; Milfont and Sibley, 2012). Existing studies show that social responsibility significantly influences individuals’ pro-environmental attitudes and behaviors (Turaga et al., 2010; Boto-García and Bucciol, 2020; Bouman et al., 2020; Jakučionytė-Skodienė et al., 2022). For instance, Jakučionytė-Skodienė and Liobikienė (2021) show that individuals’ social responsibility positively impacts their actions related to climate change mitigation. A stronger sense of social responsibility could stimulate more purchases of environmentally-friendly products (Ng and Basu, 2019). Similarly, Hwang et al. (2000) find a positive correlation between people’s sense of social responsibility and green behavioral intention. Based on the above discussions, this study assumes that individuals’ social responsibility promotes their intention to carry reusable bags for shopping. The following Hypothesis 2 is proposed:

*Hypothesis 2 (H2)*: Social responsibility has a positive impact on individuals’ intention to carry reusable bags for shopping.

### Motivations and environmentalism

Environmental and economic motivations are both vital drivers for pro-environmental attitudes and behaviors (Hage et al., 2009; Miao and Wei, 2013). As a result of severe environmental damage and growing pro-environmental activities, environmentalism has become increasingly important (Huang et al., 2014). It is found that individuals’ green purchase behavior usually depends on their environmental psychology (Schwepker and Cornwell, 1991; Young et al., 2010). According to Kang et al. (2012), consumers with a high level of environmental motivation will develop an intention to choose green hotels. Furthermore, Steinhorst and Klöckner (2018) suggest that the stimulation of environmental motivation is fundamental to achieving durable pro-environmental behaviors (Steinhorst and Klöckner, 2018). Regarding economic motivation, Cleveland et al. (2005) show that it is a significant determinant of pro-environmental behavior. Hage et al. (2009) reveal that economic motivation positively impacts people’s recycling behaviors (Hage et al., 2009). Likewise, it is reported that people’s willingness for environmental protection is boosted by economic incentives (Xu et al., 2018).

Existing studies suggest that extrinsic motivation may “crowd out,” namely weaken the effect of intrinsic motivation (Deci et al., 1999; Bowles, 2008; Luck et al., 2012), or “crowd in,” namely reinforce the effect of intrinsic motivation (Rode et al., 2015). Intrinsic motivation means that an individual engages in an activity for the inherent satisfaction it brings, or because of a personal conviction (Ryan and Deci, 2000; Authelet et al., 2021). In this study, intrinsic motivation refers to environmental motivation. Extrinsic motivation means an individual engages in an activity for its instrumental values, or economic benefits (Ryan and Deci, 2000; Authelet et al., 2021). In this study, extrinsic motivation refers to economic motivation. For instance, it is found that blood donors are motivated by moral values rather than economic benefits, and economic incentives result in a reduction of donating blood (Mellström and Johannesson, 2008). Economic incentive-based measures could crowd out voluntary pro-environmental behaviors (Turaga et al., 2010). Likewise, Rode et al. (2015) argue that the payment for environmental damage reduces people’s sense of environmental responsibility and guilt. In contrast, Xu et al. (2018) reveal that economic motivation could strengthen the power of environmental motivation for waste separation. Authelet et al. (2021) also show that economic motivation can lead to a reinforcement (“crowding-in” effect) of environmental motivation.

In terms of explaining individuals’ intention to carry reusable bags for shopping, it is interesting and remains unsolved whether economic motivation could “crowd out” or “crowd in” the effect of environmental motivation on this green behavioral intention. Thus, this study is motivated to examine how economic motivation interferes with environmental motivation regarding individuals’ intention to use reusable bags. Based on the above analysis, the following hypotheses are proposed for future examination in this study:

*Hypothesis 3 (H3)*: Environmental motivation positively impacts individuals’ intention to carry reusable bags for shopping.

*Hypothesis 4 (H4)*: Economic motivation positively impacts individuals’ intention to carry reusable bags for shopping.

*Hypothesis 5 (H5)*: Economic motivation moderates the effect of environmental motivation on individuals’ intention to carry reusable bags for shopping.

## Materials and methods

### Data collection

Using the snow-bowling sampling technique, this study conducted an online survey in November and December 2021 in China to examine the respondents’ intention to carry reusable bags for shopping. Before widely distributing the questionnaires, a pilot study of 25 respondents was conducted to ensure the statements were clear and explicit. A total of 534 questionnaires were obtained, and 532 of them were valid.

### Measures

Carrying a reusable bag for shopping was one of many sustainable actions that consumers could take to reduce usage of single-use plastics (Wang and Li, 2022). The behavior of carrying reusable shopping bags represented a sustainable and green lifestyle. The dependent variable, i.e., reusable bag using intention was constructed based on the measurement developed by Wang and Li (2022) in this study. A sample item was “I would like to bring my reusable bag to shop.” The respondents were asked to choose on a five-point Likert scale ranging from 1 (strongly disagree) to 5 (strongly agree). The Cronbach’s α of the intention to use reusable bags is 0.922.

The independent variables consisted of plastic ban awareness, social responsibility, environmental motivation and economic motivation. For each variable, the respondents were asked to rate on a five-point Likert scale ranging from 1 (strongly disagree) to 5 (strongly agree). Plastic ban awareness was measured by three items, including “I know when the plastic ban policies are issued,” “I know about the general content of the plastic ban policies,” and “I know about the specific requirements of the plastic ban policies.” The Cronbach’s α of plastic ban awareness is 0.942. Social responsibility was measured with the scale developed by Steele et al. (2008). A sample item of social responsibility is “I believe that I have a responsibility to help others.” The Cronbach’s α of social responsibility is 0.864. Regarding the motivation variables, to mitigate the intercorrelation of economic and environmental motivations and highlight the distinction between different motivations, we use the measurement questions precisely targeted to the situation of carrying reusable bags for shopping. Environmental motivation was measured by the statement “I carry a reusable bag for shopping for protecting the environment and reducing the usage of plastic bags,” and economic motivation was measured by the statement “I carry a reusable bag for shopping for saving money.” Additionally, five control variables were also incorporated regarding their influence on green bag usage, including age, gender, education, marital status and monthly income.

### Common method bias

As the data comes from a single source, the common method bias (CMB) should be concerned (Podsakoff et al., 2003). To lower the risk of CMB, following procedures were conducted. First, we ensured individuals’ anonymity, and we distributed the items for dependent and independent variables separately (Krishnan et al., 2006). Second, the Harman’s one-factor test was conducted (Harman, 1967). If one factor accounted for the majority of the covariance among the variables, CMB might exist. In this study, the un-rotated exploratory factor analysis results showed that the first factor explained less than 40% of variance, indicating no serious problem of CMB (Podsakoff et al., 2003). Third, we also examined the results for significant interactions, which were less likely to occur with CMB (Kotabe et al., 2003).

### Data analysis

Descriptive and OLS analysis was conducted to test the relationships among variables. We measured consumers’ intention to use reusable bags by a 5-point Likert scale, and the dependent variable was an average of three measuring items. Moreover, we found that the predictive variables were linearly correlated with the dependent variable. Based on the above discussion and findings, this study chose OLS to model the relationships between the dependent and independent variables. To make our model results robust, we also conducted an ordinal logistic regression to examine the influence of independent variables. The robustness check showed similar results with that of OLS and therefore confirmed the robustness of this study’s findings. However, as results of OLS were much easier to explain, we chose OLS model in our study.

## Results

### Descriptive statistics

Table 1 summarizes the descriptive statistics of the main variables of the study. Among the 532 respondents, 44.4% are male and 55.6% are female. The average age of respondents is 33. The mean of the respondents’ plastic ban awareness is 3.31, indicating a lack of awareness regarding China’s plastic ban policies. As for the respondents’ social responsibility, the mean value is 3.80. The mean values for environmental motivation and economic motivation are 4.27 and 4.17, respectively. The mean of intention to carry reusable bags for shopping is 3.96, suggesting a relatively high intention to use green bags among the respondents.

**Table 1:** Means, standard deviations, and Pearson’s correlations of the model variables (*N* = 532).

Variable	Mean	SD	1	2	3	4	5	6	7	8	9	10
1. Age	32.74	9.51	1									
2. Gender^a^	0.44	0.50	0.068	1								
3. Education	3.33	1.03	−0.248^**^	0.062	1							
4. Marital status^b^	0.53	0.50	0.539^**^	−0.039	−0.116^**^	1						
5. Income	2.66	1.59	0.151^**^	0.135^**^	0.276^**^	0.198^**^	1					
6. Plastic ban awareness	3.31	1.00	0.196^**^	0.011	−0.211^**^	0.132^**^	−0.024	1				
7. Social responsibility	3.80	0.70	0.023	0.058	0.000	−0.005	−0.049	0.331^**^	1			
8. Environmental motivation	4.27	0.99	0.030	−0.090^*^	0.069	−0.020	0.008	0.098^*^	0.162^**^	1		
9. Economic motivation	4.17	0.98	−0.063	−0.104^*^	0.043	−0.098^*^	0.009	0.045^**^	0.131^**^	0.649^**^	1	
10. Intention to carry reusable bags for shopping	3.96	0.85	0.151^**^	−0.150^**^	0.021	0.120^**^	0.094^*^	0.364^**^	0.340^**^	0.409^**^	0.323^**^	1

Reference categories: ^a^Gender = female, ^b^Material status = single.

* *p* < 0.05;

** *p* < 0.01.

Table 2 summarizes the effects of plastic ban awareness, social responsibility, environmental motivation, economic motivation, and control variables on individuals’ intention to carry reusable bags for shopping. As shown in Model 6 of Table 2, plastic ban awareness exerts a positive effect on individuals’ intention to carry reusable bags for shopping (*β* = 0.229, *p* < 0.001), supporting H1. In line with the findings of existing studies (Li and Wang, 2021; Wang and Li, 2021), the plastic ban policy is a powerful driver for green bag usage. Social responsibility significantly and positively impacts individuals’ intention to carry reusable bags for shopping (*β* = 0.247, *p* < 0.001), and H2 is supported (see Model 6). This result is consistent with previous studies that show social responsibility leads to a stronger green intention (Turaga et al., 2010; Boto-García and Bucciol, 2020; Bouman et al., 2020; Jakučionytė-Skodienė et al., 2022). This finding suggests that low social responsibility acts as a barrier to individuals’ intention to use reusable bags. Additionally, both environmental motivation (*β* = 0.280, *p* < 0.001) and economic motivation (*β* = 0.114, *p* < 0.01) have significant and positive effects on individuals’ intention to carry reusable bags for shopping (see Model 6). Echoing the findings of previous studies (Hage et al., 2009; Miao and Wei, 2013), this green behavioral intention is jointly driven by environmental and economic motivations. Thus, H3 and H4 are supported. Specifically, Model 6 shows that the intention to use reusable bags is much more stimulated by environmental motivation than by economic motivation, indicating environmentalism plays a more critical role in shaping people’s intention of green bag usage. Furthermore, as shown in Model 6 of Table 2, the interaction of environmental motivation and economic motivation shows a significantly positive effect on individuals’ intention to carry reusable bags for shopping (*β* = 0.067, *p* < 0.01). Therefore, H5 is verified. Economic motivation positively moderates the effect of environmental motivation on individuals’ reusable bag using intention.

**Table 2:** OLS regression analysis for the relationships between influential factors and individuals’ intention to carry reusable bags for shopping.

Variable		Intention to carry reusable bags for shopping						Model 1	Model 2	**Model 3**	Model 4	Model 5	Model 6
	Constant	3.376^***^ (0.203)	2.304^***^ (0.222)	1.396^***^ (0.256)	0.581^*^ (0.268)	0.440 (0.262)	0.059 (0.284)
Control variables	Age	0.014^**^ (0.005)	0.009^*^ (0.004)	0.009^*^ (0.004)	0.010^*^ (0.004)	0.008 (0.004)	0.008^*^ (0.004)
	Gender^a^	−0.296^***^ (0.074)	−0.306^***^ (0.068)	−0.332^***^ (0.066)	−0.274^***^ (0.063)	−0.258^***^ (0.062)	−0.250^***^ (0.061)
	Education	0.042 (0.038)	0.096^**^ (0.036)	0.077^*^ (0.035)	0.071^*^ (0.033)	0.053 (0.033)	0.058 (0.032)
	Marital status^b^	0.037 (0.087)	0.010 (0.081)	0.020 (0.078)	0.070 (0.074)	0.068 (0.072)	0.062 (0.072)
	Income	0.040 (0.025)	0.042 (0.023)	0.051^*^ (0.022)	0.044^*^ (0.021)	0.048^*^ (0.020)	0.046^*^ (0.020)
Independent variables	Plastic ban awareness Social responsibility Environmental motivation		0.317^***^ (0.035)	0.239^***^ (0.036) 0.317^***^ (0.049)	0.233^***^ (0.034) 0.274^***^ (0.047) 0.284^***^ (0.031)	0.222^***^ (0.033) 0.254^***^ (0.046) 0.227^***^ (0.040)	0.229^***^ (0.033) 0.247^***^ (0.046) 0.280^***^ (0.043)
	Economic motivation					0.090^*^ (0.041)	0.114^**^ (0.041)
Interaction term	Economic motivation × Environmental motivation						0.067^**^ (0.020)
R^2^		0.060	0.188	0.248	0.319	0.357	0.370
Adjusted R^2^		0.051	0.179	0.238	0.308	0.346	0.358
△R^2^		0.060	0.128	0.060	0.071	0.038	0.013
*F*-value		6.701^***^	20.308^***^	24.706^***^	30.567^***^	32.257^***^	30.638^***^

*N* = 532. Standardized regression coefficients are reported; Values in parenthesis are standard error; Reference categories: ^a^Gender = female, ^b^Material status = single.

* *p* < 0.05;

** *p* < 0.01;

*** *p* < 0.001.

As displayed in Model 6 of Table 2, age has a significant and positive effect on individuals’ intention to carry reusable bags for shopping (*β* = 0.008, *p* < 0.05). Compared with young people, elderly people prefer to carry a reusable bag for shopping, echoing the findings of Wang and Li (2021). One possible reason behind this finding is that elderly people have more time to prepare reusable bags in advance. Gender significantly and negatively affects individuals’ intention to carry reusable bags for shopping (*β* = −0.250, *p* < 0.001), indicating females are more inclined to exhibit green bag using intention compared with males. This finding is consistent with existing studies which suggest female is a more pro-environmental gender (Zelezny et al., 2000; Casey and Scott, 2006; Li et al., 2022). Based on the results of Model 6, income exerts significant and positive influence on individuals’ intention to carry reusable bags for shopping (*β* = 0.046, *p* < 0.05). Richer people exhibit stronger reusable bag using intention, suggesting that environmental motivation may play a crucial role in encouraging them to carry reusable bags for shopping. Future research is needed to explain deeper why income is positively associated with the intention to use green bags. In summary, female, older, and richer people are more inclined to carry reusable bags for shopping in China.

**Figure 1:**
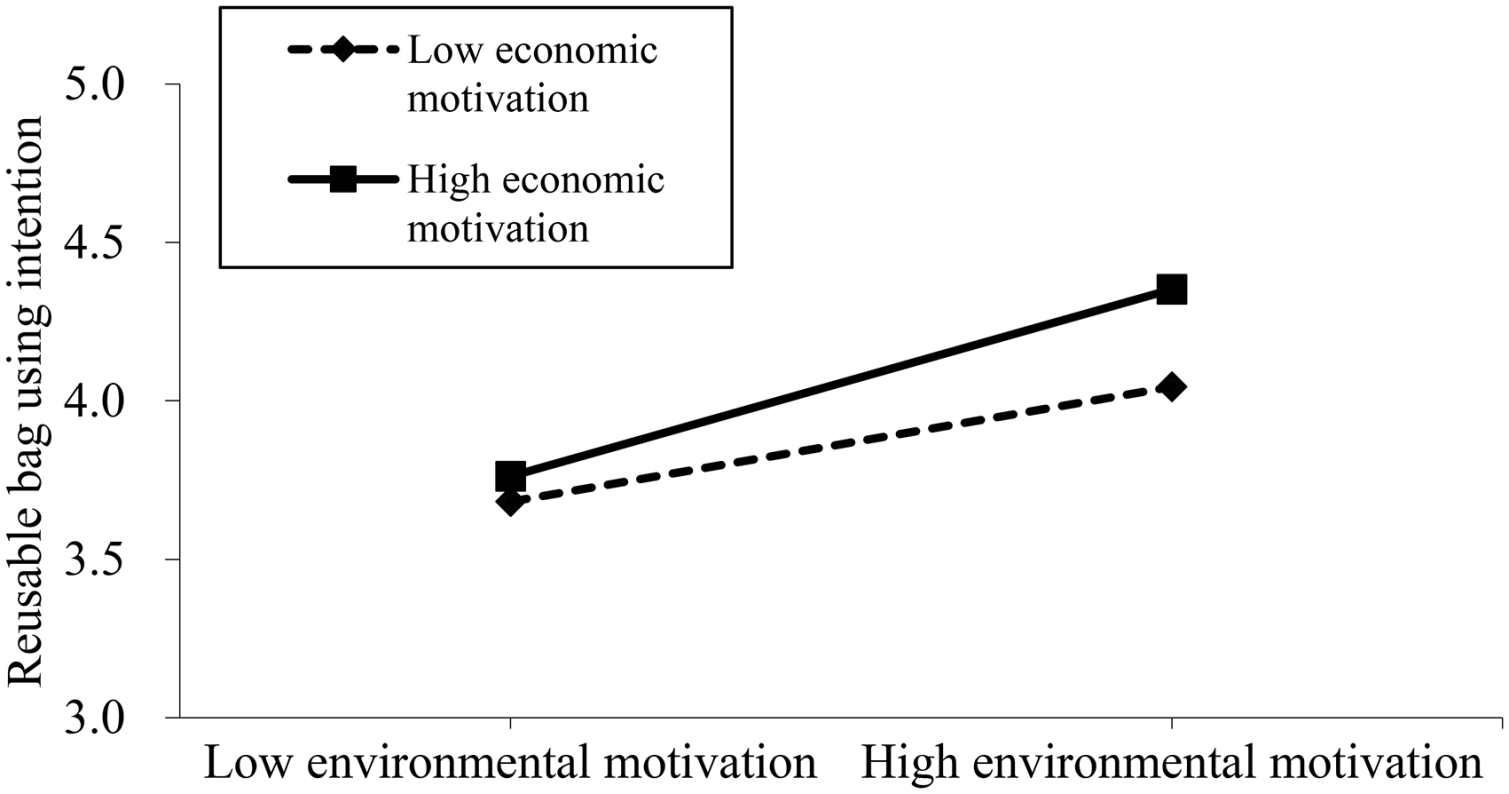
The moderating effect of economic motivation on the relationship between environmental motivation and individuals’ intention to carry reusable bags for shopping.

Figure 1 displays the moderating effect of economic motivation on the relationship between environmental motivation and individuals’ intention to carry reusable bags for shopping. As shown in Figure 1, the positive impact of environmental motivation on individuals’ intention to carry reusable bags for shopping is reinforced when economic motivation is high than when it is low. Therefore, one of this study’s contributions is the demonstration of the existence of the motivation “crowding-in” effect in explaining individuals’ intention to use reusable bags, which enlightens the government to encourage more green behavioral intention in the future through the lens of motivation.

## Discussion

The research regarding the determinants of reusable bag usage is scarce in China. To fill this research gap, this study analyzes the motivational structure behind consumers’ intention to use a reusable bags for shopping by conducting a semi-structured online survey from November to December, 2021 in China. From the above empirical analysis, it can be concluded that the intention of individual using reusable bags is significantly driven by plastic ban awareness, social responsibility, environmental motivation, and economic motivation. The novelty of this study is threefold. First, an interesting finding is a moderating effect: the higher the economic motivation the higher the impact of environmental motivation on the intention to carry reusable bags for shopping. Second, of the two motivation factors, environmental motivation has a greater impact. Third, different from the existing studies that identify the positive influence of plastic ban policies on people’s reduction of plastic bag usage (Convery et al., 2007; He, 2012; Bharadwaj et al., 2020), this study demonstrates that plastic ban awareness could promote consumers’ intention to carry reusable bags for shopping in China. This study contributes to uncovering the dynamics behind individuals’ intention to use green bags. The conclusions and implications drawn from this study are as follows:

First, the results show that plastic ban awareness positively affects individuals’ intention to carry reusable bags for shopping, which is consistent with the views of previous studies (He, 2012; Rivers et al., 2017; Bharadwaj et al., 2021). This finding contributes to a better understanding of the effectiveness of China’s plastics ban in reducing plastic pollution from a new angle of green bag usage. To get greener results, social participation is necessary and crucial, That is to say, the government, retailers and the public can all be involved in forming a sustainable shopping lifestyle in the society. First, governments’ nudging policy serves as a reminder to consumers that they could make a green choice when shopping. Rivers et al. (2017) find that Toronto’s economic nudge, i.e., plastic bag levy with $0.05 per bag, is highly effective in reducing consumers’ use of plastic disposable bags. In addition, non-economic nudging policies also contributes towards shaping consumers’ sustainable shopping style. For instance, Kaplan et al. (2018) suggest that the rise of supermarkets’ offering reusable shopping bags nudges consumers towards green consumerism. Second, many environmental messages for the detriments of plastic bags and green benefits of reusable bags could be displayed at the check-out counters in the supermarkets. In Nepal, some big supermarkets encourage the use of reusable bags as a part of their social responsibilities (Bharadwaj et al., 2021). For the convenience of consumers, they charge for the plastic bag with an option for customers to buy reusable cloth bags (Bharadwaj et al., 2021). In the future, retailers are encouraged to invest the money made from the plastic bag fee in environmental projects or to lower the cost of reusable bags (Rivers et al., 2017). Third, people’s active involvement in more green behaviors is essential and critical, such as recycling plastic bags, conducting no bag shopping, and carrying reusable bags for shopping.

Second, the results show that a higher sense of social responsibility is related to a stronger intention to carry reusable bags for shopping. This finding suggests that cultivating people’s social responsibility could promote their green behavioral intention. For the government, it is important to induce the public to take social responsibility to use reusable bags instead of plastic bags for shopping for a better environment. For the universities and colleges, this finding indicates that they could enhance environmental education by adopting a more holistic approach as social responsibility and environmental psychology are highly related and co-integrated (Bhattacharya, 2019).

Third, the results show that both environmental motivation and economic motivation positively affect the individuals’ intention to carry reusable bags for shopping, similar to the views of previous studies which underline the positive effect of motivations on green behaviors (Hage et al., 2009; Miao and Wei, 2013). Moreover, this study also finds that compared with economic motivation, environmental motivation has a greater influence on consumers’ intention to carry reusable bags. This study provides the government with a motivational insight into how to encourage consumers to use green bags. Specifically, tailored policy and managerial measures should be designed and targeted at individuals with different primary motivations. On the one hand, as the results suggested, triggering people’s environmental motivation will gain remarkable success in encouraging people to carry reusable bags for shopping. For instance, governments are suggested to publicize the detrimental effect caused by plastic bags and the green advantages of reusable bags. On the other hand, to encourage more people to carry reusable bags for shopping, the importance of economic incentives or penalties is also highlighted in this study. For instance, supermarkets are encouraged to reward consumers who carry reusable bags with a discount coupon or small gifts.

Fourth, this study demonstrates that economic motivation positively moderates the relationship between environmental motivation and the intention to carry reusable bags. In other words, this finding validates the existence of the motivation crowding-in effect in explaining individuals’ green bag using intention, echoing the existing studies about motivation crowding-in effect in predicting pro-environmental behaviors (Xu et al., 2018; Authelet et al., 2021). In addition, this finding contradicts motivation crowding-out theory’s point that economic motivation diminishes one’s environmental motivation (Turaga et al., 2010; Rode et al., 2015). This result reveals that when economic and environmental motivations are applied concurrently in the context of carrying reusable bags for shopping, the economic motivation does not reduce consumers’ environmental motivation to use green bags. The study provides important implications to better understand different motivations and their interaction to increase consumers’ green behavioral intention. The government is suggested to exert both the roles of publicity for environmental protection and the economic incentives simultaneously in the future to gain reinforced green results.

Finally, females’ intention to carry reusable bags for shopping is significantly higher than that of males, which is in line with the findings of previous research (Xiao and Hong, 2010; Hansmann et al., 2020). As for age, the results show that elderly people tend to use more reusable bags for shopping, parallel to the studies of Wang and Li (2021). In addition, compared with the low-income group, the high-income group is more prone to carry reusable bags for shopping. Taken together, female, older, and richer people are more likely to carry reusable bags for shopping in China. According to the research related to the intention to carry reusable bags for shopping, the main barrier is changing from the habit of obtaining plastic bags at supermarkets to bringing bags from home (Muralidharan and Sheehan, 2017). Other potential barriers are the extra efforts by conducting this sustainable behavior. For instance, existing studies suggest that the necessary preparation time is a key influential factor to carry reusable bags for shopping (Wang and Li, 2022). Once gotten dirty, the reusable bags also need washing efforts.

## Limitations

This study has several limitations. First, the self-reported measures for individuals’ intention to carry reusable bags for shopping may result in inaccuracy due to social desirability. Future research can investigate individuals’ actual behavior on reusable bags usage. Second, future studies are also encouraged to explain this green behavioral intention within other theoretical frameworks, such as the Theory of Planned Behavior. Additionally, this study does not investigate how social responsibility affects different types of pro-environmental attitudes and behaviors, which also deserves future research efforts.

## Data availability statement

The raw data supporting the conclusions of this article will be made available by the authors, without undue reservation.

## Ethics statement

The studies involving human participants were reviewed and approved by School of Economics and Management, Shanghai Maritime University. Written informed consent for participation was not required for this study in accordance with the national legislation and the institutional requirements.

## Author contributions

BW and YL collected the data and performed the analysis. YL and BW drafted the manuscript. YL and BW critically revised the manuscript. All authors contributed to the article and approved the submitted version.

## Conflict of interest

The authors declare that the research was conducted in the absence of any commercial or financial relationships that could be construed as a potential conflict of interest.

## Publisher’s note

All claims expressed in this article are solely those of the authors and do not necessarily represent those of their affiliated organizations, or those of the publisher, the editors and the reviewers. Any product that may be evaluated in this article, or claim that may be made by its manufacturer, is not guaranteed or endorsed by the publisher.
